# Health Outcomes of Judo Training as an Organized Physical Activity for Children and Adolescents: A Literature Review

**DOI:** 10.3390/children10081290

**Published:** 2023-07-26

**Authors:** Monika Kowalczyk, Małgorzata Zgorzalewicz-Stachowiak, Maciej Kostrzewa

**Affiliations:** 1 Department of Health Prophylaxis, University of Medical Sciences, 61-701 Poznan, Poland; neuro@ump.edu.pl; 2 Institute of Sport Science, The Jerzy Kukuczka Academy of Physical Education in Katowice, 40-065 Katowice, Poland; m.kostrzewa@awf.katowice.pl

**Keywords:** judo, children, adolescents, health benefits, physical activity

## Abstract

Judo, an organized physical activity for children and adolescents, has gained popularity worldwide. Physical activity is vital during times of rising obesity and a sedentary lifestyle in this age group. The article aims to review the current knowledge of the health benefits of judo-specific exercises for children and youth. Six international scientific databases (PubMed, Scopus, UpToDate, Web of Science, EBSCOhost, and Google Scholar) were searched from 1 January 2007 to 30 September 2022. The search focused on health-related factors regarding healthy preschool and school-aged judo practitioners. Sixteen original studies met the inclusion criteria. School-aged children aged 7–15 years were included in the analysis. The average training time was between two and six hours a week, with the judo intervention mainly ranging from nine months to three years. Most studies registered positive changes caused by judo training. The outcomes focused on maintaining or reducing body fat, increasing bone mineralization, and improving the function of the cardiorespiratory system compared to the non-practicing control group. However, there were no differences between judo and other sports groups. The main conclusions presented health benefits from judo-specific training in school-aged children and may support the World Health Organization recommendations concerning daily physical activity.

## 1. Introduction

A reduced amount of physical activity (PA) resulting from a sedentary lifestyle is one of the most alarming current problems among children and adolescents [1,2]. It is reflected in the global increase in obesity in this age group. Increased caloric intake and decreased energy expenditure from reduced PA are associated with a higher amount of adipose tissue in the body [3]. Excessive body fat (BF) during childhood and adolescence increases the occurrence of cardiovascular and metabolic risk factors, as well as the risk of adiposity and cardiovascular diseases in later life [3,4,5]. In combination with an improper diet, low PA may also contribute to decreased bone mineral density (BMD) [6,7]. It is essential during puberty, as it is assumed that the higher the bone density after puberty, the lower the risk of osteoporosis and fractures in adulthood [8].

PA in children and adolescents stimulates the development of the musculoskeletal and cardiorespiratory systems and reduces or maintains the appropriate level of BF during the development period [9,10,11]. In addition, PA has been recognized to positively affect youth’s cognitive, emotional, and social functioning [12,13]. The World Health Organization (WHO) recommends that the optimal dose of PA for health benefits in children and adolescents aged 5–17 is moderate-to-vigorous PA performed 60 min a day, on average [11]. To meet these recommendations, children and adolescents should participate in popular and accessible forms of organized PA or organized sports. Such activity is supervised by an adult and carried out according to specific rules regarding its practice [13]. The literature shows that children who participate in organized activities present a higher fitness level and more often fulfill the WHO recommendations concerning daily PA [14].

One such organized activity includes martial arts classes, such as judo. Over the years, judo has developed some competition rules and evolved into a combat sport. In 1951, the International Judo Federation (IJF) was established, and in 1964, in Tokyo, judo joined the program of the Olympic Games for the first time [15]. It has sparked interest in judo, and now there are millions of practitioners worldwide [16]. Judo is a dynamic, high-intensity sport that activates the whole body [17]. In sports competitions, to achieve success, a high level of physical fitness is required in combination with adequately developed technical and tactical skills [17,18]. Currently, judo classes are organized for preschoolers [19]. Fukuda et al. [20] claim that an early start in training better prepares for later sports competitions. In line with long-term athlete development, children around 8–9 years old start learning to train and then progress to training to train, which lasts until about 15–16 years old. Judo training for beginners mainly includes the development of general physical fitness and learning falling techniques. Moreover, ground and standing techniques are gradually introduced, followed by fighting techniques [19]. The stages of training presented by Fukuda et al. [20] also coincide with the age categories for sports competitions set by the IJF [21]. Namely, athletes aged up to 15 (U15) compete mainly at the regional and national levels. It is only later (up to 18 years—U18) that the load and intensity of the training of cadets increase. They are followed by juniors (up to 21 years old—U21) and seniors (over 21). During this period, competitors go through the stages of ‘training to compete’ and ‘training to win’ and are prepared for sports competitions at the national and international levels [20]. The stages of training according to long-term athlete development overlap with the maturation period of children and adolescents and include multi-directional development of their physical, cognitive, and emotional spheres. 

As a martial art originating in Japan and practiced by children and teenagers, judo also has educational values created by Jigoro Kano. He is the founder of judo and the first Kodokan judo school in Japan [22]. In this school, the focus, in addition to the formula of physical exercises for developing the body, is directed at improving the mind’s efficiency by applying the basic principles of Seiryoku-Zenyo (‘maximum efficiency’) and Jita-Kyoei (‘mutual prosperity’). According to Kano’s philosophy, maximum efficiency means maximizing the strength of the body and mind during judo practice and combat. On the other hand, mutual prosperity assumes that by practicing judo and self-improvement in this art, respect and discipline towards oneself and the surrounding community are developed. Sterkowicz-Przybycień et al. [23] observed a positive effect of judo on the development of social behavior in 11-year-old children who attended the training.

This study aimed to review current publications assessing the results of judo-specific training as an organized form of PA on health-related outcomes in children and adolescents at different developmental ages. To our knowledge, this is the first review of the age group up to 15 in the context of the health benefits of practicing judo.

## 2. Materials and Methods

### 2.1. Search Strategy

Articles were searched in six international scientific databases (PubMed, Scopus, UpToDate, Web of Science, EBSCOhost, and Google Scholar). Papers dating from 1 January 2007 to 30 September 2022 were considered current (from the last 15 years). When searching for relevant literature, the following terms were used: ‘judo’ in combination with ‘children’, ‘young’, ‘youth’, ‘adolescent’, ‘girl,’ and ‘boy’ and concerning ‘body composition’, ‘body fat’, ‘fat mass index’, ‘lean mass index’, ‘anthropometric features’, ‘bone mass’, ‘bone mineralization’, ‘bone health’, ‘health benefits’, ‘health outcomes’, ‘cardiorespiratory outcomes’, and ‘physical fitness’. The bibliography used in the studies included in the review was also analyzed. 

### 2.2. Selection Criteria

The selection criteria for articles were: (1) they were originally in English and published in peer-reviewed journals; (2) the participants in the experimental group were preschool and school-age children (up to 15); (3) the participants in the experimental group were a healthy population without any declared diseases; (4) the participants in the experimental group had exposure to judo-specific training; (5) the outcomes addressed health-related factors, including body composition (body fat, fat mass, lean mass), bone mineralization (bone mineral density—BMD), bone mineral content (BMC), bone area (BA) and health-related physical fitness (cardiorespiratory fitness—CRF); and (6) the study design included a control and/or reference group. 

## 3. Results

### 3.1. Study Characteristics

Ultimately, sixteen papers meeting the above criteria were included in the analyses. The youngest participants in the study were seven years old [24,25], and the oldest were 15 years old [26,27]. In four studies, their average age was up to 10, while in the remaining twelve, it was over 10. Only in Pocceco et al.’s [27] work were athletes in the reference group aged 15+ years. Nine studies included only boys, one study included only girls, and four covered both genders. In two analyzed publications, the gender structure was not provided [28,29]. Their total number was 1901, of whom 607 practiced judo. On the other hand, 1294 boys and girls qualified for the control and/or reference groups. The judo groups in the considered studies ranged from seven to 117 people, and the control or reference group size was six to 254 people. The judo group had up to 25 participants in eight studies and more than 25 in the following eight studies. The control and/or reference groups had up to 25 participants in four studies and more than 25 people in twelve. Only practitioners performing judo-specific training were included in the study group. In the reference groups, people participated in other organized activities such as recreational sporting games, track and field, soccer, karate, kung-fu, or muay thai. In Pocceco et al.’s [27] work, participants were older than the studied group but also practiced judo. The control groups consisted of people not officially participating in additional organized activities who took part only in spontaneous recreational activities and physical education classes at school.

### 3.2. Training Scenario 

The shortest training described in the analyzed papers lasted three months [30,31], and the longest lasted eight years [28]. However, in most studies, participants practiced judo for nine months (five studies) up to two (two studies) or three years (four studies). Three studies provided no information on how long the studied children had participated in judo classes. Each judo training session lasted 45 to 90 min and took place on average two to four times a week. It can translate into about two to six hours of training per week. In five articles, no training load per week was given. 

### 3.3. Body Mass Index 

Body mass index (BMI) was measured in eight studies [27,29,30,32,33,34,35,36]. In experimental, control, and/or reference groups, BMI values ranged from the 5th to the 85th percentile and were within the age norms for percentile values according to a standardized growth chart [37]. The two studies had no statistical difference in the initial-to-final measurements of BMI values. In two other studies by Triki et al. [33,34], the final BMI was statistically higher in the judo group compared to the control and soccer groups. However, the values were still within the age-appropriate percentile norms.

### 3.4. Body Composition

Changes in body composition after judo training are based on the results of thirteen studies (Table 1). In most studies (eight), body fat was assessed based on the anthropometric measurement of skin folds [24,25,26,30,32,33,38,39]. Bioelectric impedance was used in three studies [27,28,34]. Miranda et al. [35] and Missawi et al. [36] evaluated the effect of judo exercise on body composition using Dual X-ray absorptiometry (DXA). In five studies, a positive impact of judo training on body fat was found compared to control groups participating only in physical education classes [25,26,28,35,39]. One study by Pocceco et al. [27] showed that older judokas from groups U17 and 20+ had higher body fat. In three studies, the levels of lean mass in the judo groups were higher than in the control groups [32,33,36]. However, Sertic et al. [37] recorded decreased body fat after three years of intervention (measurements before and after the intervention) only in the judo group but noted no differences in the controls. Krstulović et al. [24] noticed no change in body fat after nine months of intervention but reported a significant increase in body fat in the recreational sporting games group. After a short intervention lasting only 3.5 months, Tomac et al. [30] observed no significant changes in body composition in any group. In addition, two other studies [25,34] showed no statistical changes in body composition in judo groups compared to other sports groups.

### 3.5. Bone Health

Four studies assessed the effect of judo training on bone health (Table 2) [29,31,36,40]. The method of measuring bone health was DXA. Bone densitometry assessed BMD, BMC, and BA. Ito et al. [31] registered higher BMD only in arms in the judo group compared to controls. In their following study, Ito et al. [40] also noted higher BMD in the spine, but only in males after a nine-month intervention. Costa et al. [29] also reported positive changes in BMD and BMC after intervention in the judo and muay thai groups, while the control group presented no significant change. Missawi et al. [36] similarly reported higher BMD, BMC, and BA values in the judo group in a between-group comparison.

### 3.6. Cardiorespiratory Fitness

CRF was analyzed in only three studies, and the results are presented in Table 3 [27,33,36]. Pocecco et al. [27] found that the U15 group of judokas had lower VO_2peak_ and minute ventilation than older judo groups. Lower CRF levels in younger judokas may have resulted from the possibilities and training load at this age. Missawi et al. [36] found that judokas had higher VO_2max_ than the control group. On the contrary, Triki et al. [33] found no group differences in lung volumes and capacities, VO_2max_, or VO_2_ at the ventilatory threshold. However, better results for other indices, such as respiratory exchange ratio at the ventilatory threshold, maximal respiratory exchange ratio, and heart rate, were noted in the judo group and soccer group compared to the controls. Additionally, minute ventilation was higher in judokas than in the soccer and control groups.

## 4. Discussion

The review analyzed the health benefits, especially body composition, bone health, and CRF, of judo-specific exercises in healthy children and adolescents aged up to 15. Most of the studies assessed the body composition of the study participants. Our results indicate that training positively affected body fat and lean mass in judokas compared to the age-matched control group. The methods of measuring them varied. The skin fold measurement method was often used, followed by bioelectric impedance and DXA. Only eight studies measured BMI in experimental groups. The results were within the norm, ranging from the 5th to the 85th percentiles adopted for a given age group.

In the era of growing overweight and obesity among children and adolescents, one of the essential health indicators includes body composition, which is associated with the correct amount of body fat [41,42]. It is widely observed that participation in PA may affect this indicator. Drenovatz et al. [43] showed that the participation of 7-year-old children in organized sports reduces the risk of obesity by up to 50%. Gualdi-Russo et al. [44] found that insufficient MVPA combined with sedentary behavior increases fat mass in children and adolescents. In another review, García-Hermoso et al. [45] showed that particularly vigorous activity positively affected overall adiposity and suggested that this type of PA should be recommended for children and adolescents. Judo training can thus be offered to schoolchildren. It includes moderate- to high-intensity elements, thanks to which children can develop general fitness and learn judo techniques by performing vigorous exercises, such as speed and endurance [20,46]. In addition to the level of effort during judo training, authors pay particular attention to the time of intervention and training load per week, which may significantly impact changing body composition [47]. In our review, judo training for schoolchildren lasted from 45 to 90 min and was repeated two to four times a week, which translates into about two to as many as six hours of additional organized PA per week. Another assessed element was the period of observations, which translates into the health benefits of judo training. Tomac et al. [30] described a minimal intervention time amounting to 3.5 months of judo training and observed no changes in body composition. Only in interventions lasting at least nine months, and even two to three years, were positive changes in body fat and lean mass noted. It is worth noting that the effects of sports activity on body composition are not always noticeable in this age group [47,48,49]. It is observed that, in addition to the type of training and the training load itself, the biological activity of sex hormones during puberty begins to significantly affect the level of body fat. Based on the body fat analysis of Australian children and adolescents, Telford et al. [50] found that between the ages of 12 and 16, body fat increased in girls and decreased in boys. Generally, girls maintain a higher body fat level during puberty and adulthood than boys.

In the growing bodies of children and adolescents, intensive bone growth occurs. Additionally, puberty is considered important for adequate bone mass accrual, which helps prevent osteoporosis in the future [7]. In addition to genetic factors, PA and a proper diet are crucial to ensuring bone health [8]. Janssen et al. [9] analyzed children aged 5–17 who engaged in PA. They found that ten-minute moderate-to-intense exercises performed two to three times a week in combination with aerobic weight-bearing exercises had a beneficial effect on bone development. Tan et al. [51] presented similar findings based on a review of 37 studies, including respondents aged 5 to 18. They found that weight-bearing exercises had a positive effect on bone strength. The changes concerned mainly bone structure rather than bone mass. Weight-bearing exercises are also part of judo training. In addition, the specificity of judo is related to the high intensity of the activity itself. Resistance exercises in contact with the opponent and frequent falls on the mat can benefit bone health [17,52]. Agostinete et al. [47] observed a beneficial effect of judo-specific exercises in the 10–17 age group on the whole-body areal bone mineral density (aBMD) compared to those trained in swimming. In addition, the aBMDs of the upper limbs were also higher in judokas when compared to those who practiced karate, kung-fu, or swimming. Our results of bone densitometry measured with DXA in the groups practicing judo showed beneficial changes in the bone structure after the intervention compared to controls. It mainly concerns changes in arms. It may be related to the specificity of judo training, during which various judogi grips and multiple falls on the mat are performed.

The assessment of health-related PA in children and adolescents also includes CRF. Among its beneficial effects described in the literature is the reduced risk of cardiovascular diseases [53,54]. Telford et al. [50] showed that a higher level of PA resulted in higher CRF values in children and adolescents participating in organized club sports activities compared to those who did not exercise. Judo-specific exercises for schoolchildren may affect CRF. Triki et al. [33] and Missawi et al. [36] observed better cardiorespiratory indices in judo trainees than controls. Pocceco et al.’s [27] results indicate that CRF develops in young people with increasing age and training load. García-Hermoso et al. [45] suggested that CRF positively correlated with vigorous PA and outperformed moderate PA. These authors observed that higher PA levels increase the availability and use of oxygen in the body, so the trainee better adapts to the effort. Fukuda et al. [18] indicate that judo training influences the development of CRF, which may improve the ability to sustain high-intensity exercise, delay the onset of fatigue, and improve the body’s recovery capacity by lowering the maximum heart rate and enhancing lactate utilization. The above studies suggest that training and PA may positively affect CRF in young judokas. However, the effects may differ depending on the measured variables and the athletes’ age and training intensity.

Our review had some limitations. The majority of respondents in the judo group were boys. It would be interesting to observe the health benefits of judo in comparable groups consisting of only girls or both genders. Moreover, in the included studies, heterogeneity was observed in the number of group participants and the duration and frequency of judo training. In addition, various research methods were used to assess body composition, bone health, and CRF. Unifying research techniques and intervention methods would help indicate the maximum amount of judo training that has the best health effects on schoolchildren. The analysis of physical outcomes should be extended to include the assessment of nutrition or sleep quality, as these factors may also affect health indicators. The control group in most analyzed studies consisted of people attending only compulsory physical education classes in schools, which resulted in poorer health effects. In the future, it is worth examining the health benefits of judo training compared to other organized sports activities, primarily from a long-term perspective.

Despite the limitations described, our study confirms favorable health outcomes in judo practitioners aged up to 15. Additionally, the multidimensionality of health benefits found even in youth will certainly affect the career development of future professional judo athletes.

## 5. Conclusions

The review found that organized judo classes had positive health outcomes for school-aged children. The beneficial changes concerned body fat, lean mass, bone health, and CRF. They were found after at least nine months of intervention. These health benefits significantly impact the current and future health of children aged 7–15 who undergo intensive developmental changes in the body. Therefore, long-term studies should be conducted on the health-related outcomes of judo training for boys and girls using unified and standardized research techniques. In addition, further research should determine the optimal dose and training load of judo to bring health benefits to participants in this age group, considering the WHO recommendations regarding PA.

## Figures and Tables

**Table 1:** Body composition measurement.

Author(s) (Year)	Sample n (Sex); Age (Average or Range)	BMI M ± SD (kg/m^2^)		Measurement Characteristics	Judo (Frequency, Dosage and/or Duration)	Reference and/or Control (Frequency, Dosage and/or Duration)	Outcomes (Significant Differences)
		Judo	Reference and/or Control				
Sertic et al. (2009) [38]	87 (100% m); 11 y J n = 17, C n = 70	NA	NA	Skinfold	3-y program **	C: only physical education classes **	In J group BF ↓ after intervention. No differences in between-group comparison.
Drid et al. (2009) [26]	371 (100% m); 11–15 y J n = 117, C n = 254	NA	NA	Skinfold	J: 2 times/w for 2 y	C: only physical education classes **	J group presented ↓↓ BF compared to C.
Krustolovic et al. (2010a) [24]	79 (100% f); 7 y J n = 30, RSG n = 49	NA	NA	Skinfold	1 session (0.75 h), 3 times/w for 9 m	RSG: 1 session (0.75 h), 3 times/w for 9 m	No change in BF in the J group after intervention; in the RSG group BF ↑.
Krustolovic et al. (2010b) [25]	202 (100% m); 7 y J n = 41, TF = 68, F n = 38, C n = 55	NA	NA	Skinfold	1 session (0.75 h), 3 times/w for 9 m	TF or S: 1 session (0.75 h), 3 times/w for 9 m, C: only physical education classes: 1 session (0.75 h), 3 times/w for 9 m	J, TF, and S groups had ↓↓ BF compared to C in Fin measurement.
Triki et al. (2012) [32]	96 (100% m); 11 y J n = 32, F n = 32, C n = 32	Fin: 18.5 ± 2.2 *	F Fin: 16.9 ± 1.4 * C Fin: 16.6 ± 1.7 *	Skinfold	6–8 h/w for min. 3 y	S: min. 3 y for 6–8 h/w, C: 2 or fewer h/w of physical activity at school **	J and S groups presented ↑↑ LM compared to C. Only S group had ↓↓ BF than C.
Triki et al. (2013) [33]	96 (100% m); 11 y J n = 32, F n = 32, C n = 32	Fin: 18.4 ± 2.2 *	F Fin: 17.4 ± 1.6 * C Fin: 17.3 ± 2.1 *	Skinfold	1 session (1.5 h), 4 times/w for min. 3 y	S: 1 session (1.5 h), 4 times/w for min. 3 y C: 2 or fewer h/w of physical activity at school **	J and S groups presented ↑↑ LM compared to C. Only S group had ↓↓ BF than C.
Protic-Gava et al. (2019) [39]	148 (100% m); 13 y J n = 58, C n = 90	NA	NA	Skinfold	2 h/w for min. 3 y	C: only physical education classes **	J group presented ↓↓ level of BF in the between-groups comparison.
Tomac et al. (2020) [30]	45 (21 m); 8–9 y J n = 22, C n = 23	In: 18.2 ± 4.3 Fin: 17.8 ± 4.0	In: 17.4 ± 4.8 Fin: 18.1 ± 3.5	Skinfold	3.5 m **	C: only physical education classes **	No change in BF in both groups after intervention and in between-group comparison.
Pocecco et al. (2012) [27]	25 (100 % m); 12+ y J U15 n = 7, J U17 n = 10, J +20 n = 8	J U15 Fin: 17.9 ± 1.6 *	J U17 Fin: 21.1 ± 0.7 * J +20 Fin: 26.6 ± 3.7 *	BIA	U15 1.5 h training session **	J U17 or J +20 1.5 h training session **	J +20 group has ↑↑ BF when compared to J U15 and J U17.
Pion et al. (2014) [34]	30 (100% m); 12 y J n = 10, K n = 9, T n = 11	J Fin: 17.3 ± 1.9	K Fin: 16.1 ± 1.4 T Fin: 17.6 ± 2.2	BIA	NA **	NA **	No group differences.
Lech et al. (2020) [28]	154 ^a^; J 1; n = 21 (11–12 y), C n = 71 J 2; n = 18 (13–14 y), C n = 44	NA	NA	BIA	1–8 y of training experience **	C: no formal physical activities **	J groups (1 and 2) noted ↓↓ values in BF compared to both C groups.
Miranda et al. (2017) [35]	105 (68 m); 10 y J n = 65, C n = 40	In: 19.9 ± 4.8 Fin: NA	In: 19.9 ± 4.6 Fin: NA	DXA	2 h/w for 9 m	C: no formal physical activities **	J group had ↓↓ BF compared to C in Fin measurement.
Missawi et al. (2018) [36]	119 (100% m); 11 y J n = 50, C n = 69	Fin: 17.7 ± 3.8	Fin: 17.7 ± 3.3	DXA	3–6 h/w for min. 2 years	C: only physical education classes for 2 times to 1 h/w **	J group presented ↑↑ LM in arms and legs in comparison to C.

Legend: ^a^ Sex not reported; * Significant difference between groups (*p* < 0.05); ** Frequency, dosage, and/or duration not reported; m, male; f, female; h, hour/s; w, week/s; m, month/s; y, year/s; min., minimum; J, judo; S, soccer; RSG, recreational sporting games; TF, track and field; K, karate; C, control; NA, not available; BIA, bioelectric impedance; DXA, dual X-ray absorptiometry; In, initial; Fin, final; ↓, decreased; ↑, increased; ↑↑, higher; ↓↓, lower; BF, body fat; LM, lean mass.

**Table 2:** Bone mineral density measurement.

Author(s) (Year)	Sample n (Sex); Age (Average or Range)	BMI M ± SD (kg/m^2^)		Measurement Characteristics	Judo (Frequency, Dosage and/or Duration)	Reference and/or Control (Frequency, Dosage and/or Duration)	Outcomes (Significant Change)
		Judo	Reference and/or Control				
Ito et al. (2016) [31]	137 (83 m); 13 y J n = 17, K n = 14, KF n = 16 C n = 90	NA	NA	DXA	6.4 ± 5.4 h/w for min. 3 m	K: 10.5 ± 2.8 h/w for min. 3 m KF: 3.2 ± 1.2 h/w for min. 3 m C: no formal physical activities **	BMD of J group in arms was ↑↑ than only C.
Ito et al. (2017) [40]	79 (44 m); 13 y J n = 21, K/KF n = 29, C n = 29	NA	NA	DXA	8 h/w for 9 m	K/KF: 8 h/w for 9 m C: recreational activities no more than twice a w **	BMD in males of J group in spine was ↑↑ than in males of C.
Costa et al. (2018) [29]	32 ^a^; 11 y J n = 17, MT n = 9, C n = 6	In: 19.1 ± 4.8 Fin: NA	MT In: 18.7 ± 3.6 Fin: NA C In: 20.3 ± 4.3 Fin: NA	DXA	3 h/w for 9 m	MT:1 session (1.5 h), 2 times/w for 9 m C: no formal physical activities **	In J group BMC ↑ in arms, trunk, pelvis, and total in between In and Fin measurements. MT group BMD ↑ in legs and column and BMC ↑ in trunk in between In and Fin measurements.
Missawi et al. (2018) [36]	119 (100% m); 11 y J n = 50, C n = 69	Fin: 17.7 ± 3.8	Fin: 17.7 ± 3.3	DXA	3–6 h/w for min. 2 y	C: only physical education classes for 2 times to 1 h/w **	BMD, BMC, and BA were ↑↑ in the J group in the between-groups comparison for the whole body index, mainly for both dominant and non-dominant arms, hips, legs, and whole radius.

Legend: ^a^ Sex not reported; ** Frequency, dosage, and/or duration not reported; m, male; h, hour/s; w, week/s; m, month/s; y, year/s; min., minimum; J, judo; K, karate; KF, kung-fu; MT, muay thai; C, control; NA, not available; DXA, dual X-ray absorptiometry; In, initial; Fin, final; ↑, increased; ↑↑, higher; BMD, bone mineral density; BMC, bone mineral content; BA, bone area.

**Table 3:** Cardiorespiratory fitness measurement.

Author(s) (Year)	Sample n (Sex); Age (Average or Range)	BMI M ± SD (kg/m^2^)		Measurement Characteristics	Judo (Frequency, Dosage and/or Duration)	Reference and/or Control (Frequency, Dosage and/or Duration)	Outcomes (Significant Change)
		Judo	Reference and/or Control				
Pocecco et al. (2012) [27]	25 (100 % m); 12+ y J U15 n = 7, J U17 n = 10, J +20 n = 8	J U15 Fin: 17.9 ± 1.6 *	J U17 Fin:21.1 ± 0.7 * J +20 Fin: 26.6 ± 3.7 *	Physiological tests (arm crank and leg cycling ergometer)	U15 1.5 h training session **	J U17 and J +20 1.5 h training session **	U15 J group presented ↓↓ VO_2peak_ and V_E_ in both tests of arm crank and leg cycling ergometer compared to older J groups.
Triki et al. (2013) [33]	96 (100% m); 11 y J n = 32, F n = 32, C n = 32	Fin: 18.4 ± 2.2 *	F Fin: 17.4 ± 1.6 * C Fin: 17.3 ± 2.1 *	Physiological tests (cyclo-ergometer exercises)	1 session (1.5 h), 4 times/w for min. 3 y	S: 1 session (1.5 h), 4 times/w for min. 3 y C: 2 or fewer h/w of physical activity at school **	RER(VT) and RER_max_ in J and S groups were ↑↑ than C. V_E_ was ↑↑ than S and C. HR(VT) was slower in the J and S groups than in the C group.
Missawi et al. (2018) [36]	119 (100% m); 11 y J n = 50, C n = 69	Fin: 17.7 ± 3.8	Fin: 17.7 ± 3.3	Maximal aerobic power (20-meter shuttle run test of Leger)	3–6 h/w for min. 2 y	C: only physical education classes for 2 times to 1 h/w **	J group had ↑↑ VO_2max_ compared to the C group.

Legend: * Significant difference between groups (*p* < 0.05); ** Frequency, dosage, and/or duration not reported; m, male; h, hour/s; w, week/s; y, year/s; min., minimum; J, judo; S, soccer; C, control; Fin, final; ↑↑, higher; ↓↓, lower; VO_2peak_, peak oxygen consumption; VE, minute ventilation; RER(VT), respiratory exchange ratio at ventilatory threshold; RERmax, maximum respiratory exchange ratio; HR(VT) heart rate at ventilatory threshold; VO_2max_, maximal oxygen consumption.

## Data Availability

Data used in this study are presented within the manuscript.
